# 853. Rapid, non-invasive detection of *Borrelia miyamotoi* infection using a plasma-based microbial cell-free DNA sequencing test

**DOI:** 10.1093/ofid/ofad500.898

**Published:** 2023-11-27

**Authors:** Sharjeel Ahmad, Douglas Kasper, Luis Rubio, Javier E Marinez

**Affiliations:** University of Illinois College of Medicine-Peoria, Peoria, Illinois; University of Illinois College of Medicine, Peoria, IL; UCSF, San Francisco, CA; Infectious Disease Consultants, Apopka, Florida

## Abstract

**Background:**

*Borrelia miyamotoi* is a zoonotic infection transmitted by the same genus of ticks that transmits *Borreliella burgdorferi* which causes Lyme disease. It can cause an acute non-specific febrile illness ("hard tick-borne relapsing fever") with chills, sweats, headache, neck stiffness, fatigue, myalgias, and arthralgias. Serious manifestations such as meningitis or meningoencephalitis or uveitis can occur in immunocompromised hosts. Many features of this infection are indistinguishable from other tick-borne illnesses. Rapid, non-invasive diagnosis of *B. miyamotoi* by microbial cell-free DNA (mcfDNA) sequencing of plasma offers a means to overcome these limitations.

**Methods:**

The Karius Test^TM^ (KT) detects and quantifies mcfDNA in molecules/µL (MPM) from > 1000 organisms in plasma (performed at the CLIA certified/CAP accredited Karius laboratory). KT detections of *B. miyamotoi* were compiled from three medical centers in different regions of United States. Clinical review was performed by the health care providers.

**Results:**

KT detected *B. miyamotoi* in three patients. All were adult males in whom the diagnosis was unexpected. None of the patients recalled tick bites. (See Table 1 for patient characteristics). Two patients were on immunomodulator therapy while the third one had hepatitis C (treated) – mediated cirrhosis. A broad infectious disease work-up was performed and broad-spectrum empiric antibiotic was initiated in one case. KT was the first test to identify *B. miyamotoi* as the microbiological diagnosis in all cases and was the only test to establish the diagnosis in two cases, with the third having subsequent positive *Borrelia* PCR testing. All responded well to treatment with doxycycline with resolution of symptoms and lab abnormalities.

**Figure:**
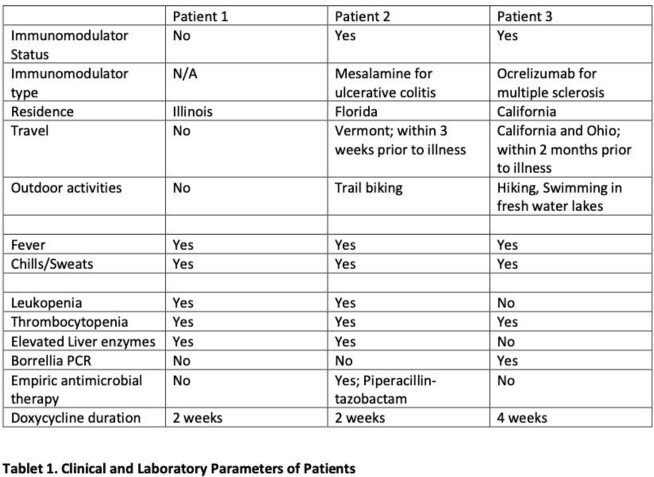

**Conclusion:**

KT enabled rapid, non-invasive, plasma-based diagnosis of "hard tick-borne relapsing fever" caused by *B.miyamotoi*. Given an unclear etiology and a potentially diverse differential diagnosis, especially in immunocompromised patients, an unbiased diagnostic test such as the KT can prove a useful complement to conventional diagnostic work-up to provide real-time results. Additionally, mcfDNA sequencing can prove useful in detecting emerging, diagnostically challenging microbes.

**Disclosures:**

**All Authors**: No reported disclosures

